# A detailed examination of the worldwide impact of type 2 diabetes linked to dietary risks: insights from the Global Burden of Disease Study (1990–2021)

**DOI:** 10.3389/fendo.2026.1701350

**Published:** 2026-02-20

**Authors:** Hui Wang, Linhan He, Xiaocui Wang, Yingxuan Du, Zhilian Mu, Ying Shi, Yuan Wang, Qiao Qiao, Qiang Tong, Hongting Zheng

**Affiliations:** Department of Endocrinology, Metabolic and Chronic Disease Science Innovation Center, The Second Affiliated Hospital of Army Medical University, Chongqing, China

**Keywords:** dietary risk, global burden of disease, prevention strategies, public health, type 2 diabetes mellitus

## Abstract

**Objective:**

Type 2 diabetes mellitus (T2DM) is a considerable public health concern worldwide. In this study, the impact of T2DM attributable to dietary risk (T2DM-ADR) on disability-adjusted life years (DALYs) and deaths was analyzed using global burden of disease (GBD) data from 1990 to 2021 across 204 countries.

**Methods:**

T2DM-ADR was analyzed in 204 different countries/regions from 1991 to 2021. Key indicators included DALYs and death tolls, which were assessed according to age, sex, and sociodemographic index (SDI). This study used descriptive and trend analyses, along with the ARIMA model, to predict future outcomes. Trends were quantified using age-standardized rates (ASRs), and the estimated annual percentage change (EAPC) in the variables was used to quantify them.

**Results:**

DALYs due to dietary risk increased from 6,450,217 in 1990 to 19,146,810 in 2021, with the ASR increasing from 159.96 to 221.34 per 100,000. Deaths increased from 164,060 to 381,416, but age-standardized death rates somewhat decreased. China, India, and the U.S. reported the highest T2DM-ADR burdens, with a 260% increase in DALYs in middle-SDI regions, while low-SDI regions had the highest ASR. The 65–69 age group showed a significant increase in DALYs, with males surpassing females in 2021. Predictions for 2050 suggest that global DALYs will reach 17,865,944 for males and 18,121,264 for females, with deaths estimated at 264,822 for males and 305,383 for females, along with increasing ASR.

**Conclusions:**

This study highlights the considerable influence of dietary risk on the global prevalence of T2DM based on the GBD database. There is an urgent need to improve global dietary habits, health education, and food policy regulations to reduce the impact of T2DM on public health.

## 1 Background

Type 2 diabetes mellitus (T2DM), a prevalent chronic metabolic disorder, has been recognized as among the most significant public health challenges of the 21st century. According to the latest projections from the International Diabetes Federation (IDF), the global number of people with diabetes is expected to reach 780 million by 2045 (1, 2). T2DM not only significantly impairs quality of life but also substantially increases the risk of cardiovascular diseases, chronic kidney disease, and certain types of cancers. These factors place substantial burdens on both individual health and socioeconomic systems (3–6).

T2DM incidence is the combined effect of genetic predisposition and environmental exposures, such as diet, physical activity, and lifestyle patterns. Dietary factors, which are important and adjustable, play key roles in the onset and progression of T2DM (7). The acceleration of urbanization and globalization has significantly altered dietary patterns, leading not only to a general shift away from traditional, healthy diets but also specifically to the increasing prevalence of diets high in sugar and fat yet low in fiber. Dietary patterns have significantly changed because of the acceleration of urbanization and globalization, which has also caused high-sugar, high-fat, and low-fiber foods to gradually replace conventional, healthy dietary patterns. These unhealthy dietary habits not only lead to the prevalence of obesity and metabolic syndrome but also directly increase the risk of T2DM (7, 8). Synthesizing evidence from 155 controlled intervention studies (n=5,086), a systematic review and meta-analysis indicated that the consumption of fructose-containing sugars in hypercaloric diets adversely affects fasting insulin levels, contributing to the development of insulin resistance and impaired glycaemic regulation (9).

Another prospective cohort study in U.S. adults reported a risk ratio of 1.62 for red meat and a risk ratio of 1.51 for processed meat for the risk of T2DM, reflecting their higher risk (10). As a complementary finding, a web-based study revealed that increased intake of fiber, whole grains, fruit, and vegetables is inversely associated with the risk of T2DM, highlighting the importance of diet composition in prevention strategies (11). Although many studies on the association between diet and T2DM have been reported, global trends of T2DM attributable to dietary risk (T2DM-ADR) need more detailed examination (8). The use of region-specific data has, in turn, generated fragmented evidence and, as such, provides an incomplete picture of the overall, population-level patterns and spatial–temporal trends of T2DM-ADR.

This study provides an overview of the global burden of T2DM-ADR and is based on Global Burden of Disease (GBD) 2021 data for the world and 204 countries and territories from 1990 to 2021. Major metrics (disability-adjusted life years, deaths, years of life lost, and years lived with disability) were summarized for children and adults by age, sex, SDI and region. Our study revealed a substantial and growing global burden, reflecting considerable inequality across these demographic and socioeconomic factors. The major contributions of this work are integrated stratified analysis and time-series forecasting; the former enables the identification of high-risk groups and future trends. These findings provide critical evidence to guide targeted prevention strategies and inform strategic, long-term health planning to reduce the global burden of T2DM-ADR.

## 2 Methods

### 2.1 Data source and extraction

The focus of this study was the burden of T2DM-ADR. The data were obtained from the GBD Study 2021 using the GBD Results Tool on the Global Health Data Exchange (GHDx) platform (https://vizhub.healthdata.org/gbd-results/).

The search parameters included the following: 1) Cause: “Type 2 diabetes mellitus”, 2) Risk: “Dietary risks”, 3) Measures: “DALYs (Disability-Adjusted Life Years): Disability adjusted life year is a measure of overall disease burden, expressed as the cumulative number of years lost due to ill health, disability or early death”, “deaths”, “YLLs (Years of Life Lost): Years of life lost due to premature mortality”, “YLDs: Years Lived with Disability”, 4) Location: “all locations”, 5) Years: “1990–2021”, 6) Metrics: “number, rate, and percentage”, 7) Sex: “male, female, and both”, and 8) Age: “age-standardized, all ages, and corresponding 5-year bands”.

### 2.2 Descriptive analysis

We conducted a descriptive analysis to characterize the distribution of T2DM-ADR across multiple dimensions. The burden was described using key metrics, including DALYs, deaths, YLLs, YLDs, and their corresponding age-standardized rates (ASRs), along with corresponding 95% uncertainty intervals (UIs). To ensure granularity and comparability, the burden was evaluated across multiple stratifications: at the global level, by SDI quintiles, by GBD regions, and by 5-year age groups and sex. SDI is a composite metric that synthesizes a region’s average income per capita, educational attainment levels, and fertility rates into a single value, serving as a comprehensive gauge of its overall socioeconomic development status. For this study, countries and regions were categorized into quintiles based on their SDI values: low (SDI <20th percentile), low-middle (20th–39th), middle (40th–59th), high-middle (60th–79th), and high (SDI≥80th percentile).

### 2.3 Trend analysis

To quantify the temporal trends in the burden of T2DM-ADR from 1990 to 2021, we conducted a multilevel analysis. In addition to examining the overall patterns at the global level, we performed stratified analyses to identify disparities. Specifically, trends were assessed according to different levels of socioeconomic development, as defined by SDI quintiles, and across geographic regions classified by GBD. Furthermore, age- and sex-specific trends were evaluated by stratifying the data into 5-year age groups and by sex.

### 2.4 Predictive analysis

To forecast the future burden of T2DM-ADR from 2030 to 2050, we employed time-series forecasting models. We constructed and fitted both autoregressive integrated moving average (ARIMA) and exponential smoothing (ES) models to historical data (1990–2021). These models integrated key predictive variables, including demographic data, SDI, and major dietary risk factors. Population projections for future periods were used to calibrate crude estimates of DALYs and deaths, thereby accounting for expected shifts in population size and demographic structure. To quantify the differential impact of dietary risk, the models generated forecasted values of T2DM-ADR incidence, DALYs, and deaths, stratified at the global, regional (SDI and GBD), and demographic (age-sex) levels.

### 2.5 Statistical metrics and tools

To ensure comparability across populations with differing age structures, the disease burden was primarily expressed as an ASR. The calculation of the ASR was performed with reference to the previous literature (12). The estimated annual percentage change (EAPC) is a widely used metric that models the underlying smooth trend in age-standardized rates over time. The EAPC and its 95% CI were calculated as follows: EAPC = 100 × (exp(β) – 1), where β is the coefficient of the time variable in the regression model. A positive EAPC value indicates an increasing trend over the study period, whereas a negative value signifies a decreasing trend. All statistical analyses and visualizations were conducted using R software (version 4.4.2), with the significance level set at 0.05.

## 3 Results

### 3.1 Changes in the DALYs of T2DM-ADR

**Figure 1:**
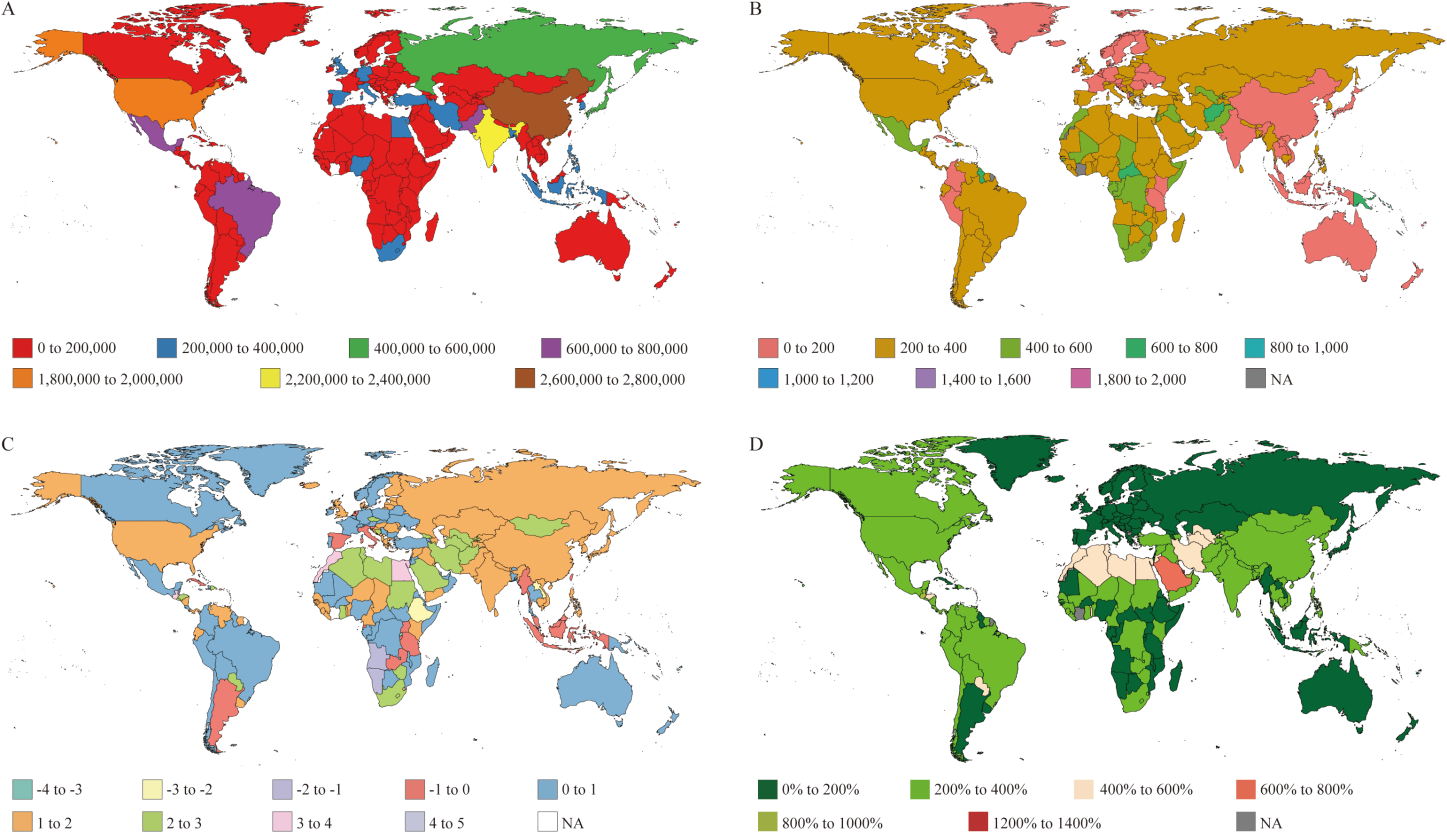
DALYs of T2DM attributable to dietary risks from 204 countries. **(A)** Number of DALYs in T2DM-ADR patients. **(B)** Age-standardized rates of DALYs in 2021. **(C)** EAPC map of age-standardized DALY rates of T2DM-ADR. **(D)** Changes in the number of DALYs.

The impact of dietary risk on T2DM has increased worldwide, with the number of disability-adjusted life-years (DALYs) lost. In 1990, 6,450,217 DALYs were due to T2DM (95% UI: 1,256,122–10,613,945). By 2021, this number had risen markedly to 19,146,810 (95% UI: 4,147,239–31,937,618) (see **Figure 1A**, **Table 1**). After adjustment for age, the number of DALYs per 100,000 people increased from 159.96 in 1990 to 221.34 in 2021 (EAPC 1.59) (**Figure 1C**, **Table 1**). YLLs were the leading contributor to DALYs due to T2DM in 1990, but YLDs were the leading contributor in 2021 (see **Supplementary Figure 1** and **Supplementary Tables 1**, **2**).

**Table 1:** DALYs of T2DM Between 1990 and 2021 at the Global and Country-Level.

Location	1990 DALYs (95% UI)	1990 ASR (95% UI)	2021 DALYs (95% UI)	2021 ASR (95% UI)	EAPC_CI
Global	6,450,217 (1,256,122-10,613,945)	159.96 (31.17-262.82)	19,146,810 (4,147,239-31,937,618)	221.34 (47.97-368.92)	1.59 (1.37-1.81)
People’s Republic of China	769,703 (99,186- 1,395,086)	85.11 (10.99-153.98)	2,702,413 (337,623-4,927,021)	133.19 (16.98-244.54)	2.5 (2.21-2.8)
United States of America	663,227 (149,822-1,054,918)	217.72 (49.39-345.99)	1,993,463 (537,271-3,239,962)	371.91 (101.84-602.74)	1.99 (1.53-2.44)
India	626,542 (135,796-1,076,371)	129.67 (27.66-223.58)	2,393,928 (588,406-4,005,473)	194.55 (47.19-325.89)	2.03 (1.47-2.6)
Niue	13 (2-23)	620.62 (94.31-1096.73)	21 (4-36)	968.74 (192.59-1711.31)	1.8 (1.53-2.07)
Tokelau	9 (1-15)	650.86 (88.63-1134.23)	12 (2-21)	805.96 (142.47-1434.09)	1.04 (0.87-1.21)
Fiji	6,261 (805-10,602)	1564.95 (192.91-2655.79)	16,164 (2,592-27,913)	1978.51 (322.59-3413.58)	1.51 (0.93-2.09)
Trinidad and Tobago	8,517 (2,333-13,588)	1000.11 (272.52-1597.98)	18,220 (5,156-30,739)	947.99 (271.66-1595.52)	0.55 (0.25-0.85)
Marshall Island	160 (19-282)	891.36 (99.58-1585.17)	602 (86-1,084)	1451.00 (196.04-2622.39)	2.73 (1.76-3.72)
Democratic People’s Republic of Korea	9,732 (1,624-17,749)	55.93 (9.37-101.70)	30,056 (5,551-52,925)	89.72 (16.71-157.68)	2.33 (2.15-2.5)

However, the global distribution of DALYs due to T2DM-ADR is not uniform. China had the highest number of DALYs attributed to T2DM-ADR in 2021, with 2,702,413 DALYs (95% UI: 337,623–4,927,021), up from 769,703 DALYs (95% UI: 99,186–1,395,086) in 1990, representing an EAPC of 2.5. For DALYs, the contribution of YLDs was greater than that of YLLs, whether in 1990 or 2021. In 1990, the United States of America ranked second, with 663,227 DALYs (95% UI: 149,822–1,054,918), but by 2021, the number of DALYs had decreased to the third position, with 1,993,463 DALYs (95% UI: 537,271–3,239,962). YLLs were predominant in 1990, whereas YLDs were predominant in 2021. India’s T2DM-related DALYs increased from 626,542 (95% UI: 135,796–10,763,710) in 1990 to 2,393,928 (95% UI: 588,406–4,005,473) in 2021, ranking second globally, with an EAPC of 2.03. YLLs were the main contributors in India in both 1990 and 2021 (**Figure 1**, **Table 1**, **Supplementary Tables 1**, **2**).

In terms of age-standardized DALY rates, Fiji had the highest rates: 1,565 (95% UI: 193–2,656) per 100,000 in 1990, which increased to 1,978.5 (95% UI: 322.59–3,413.58) per 100,000 in 2021. Moreover, the Democratic People’s Republic of Korea recorded the lowest numbers: 55.9 (95% UI: 9.4–101.7) per 100,000 in 1990, which increased to 89.72 (95% UI: 16.71–157.68) per 100,000 in 2021 (**Figure 1**, **Table 1**).

### 3.2 Trends in deaths from T2DM-ADR

**Figure 2:**
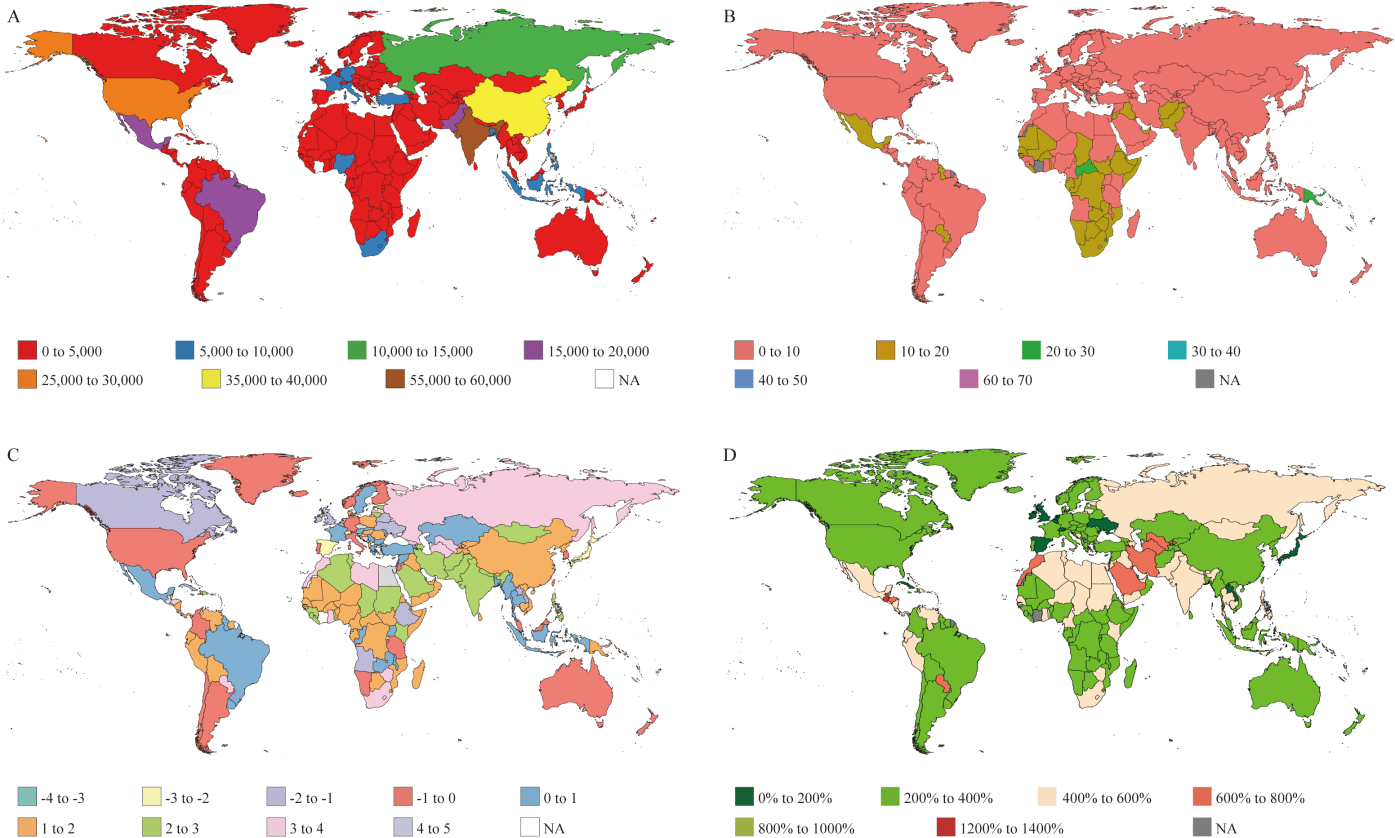
Deaths of T2DM attributable to dietary risks from 204 countries. **(A)** Number of deaths in the T2DM-ADR cohort. **(B)** Age-standardized rates of death in 2021. **(C)** EAPC map of age-standardized death rates of T2DM-ADR patients. **(D)** Changes in the number of Deaths.

From 1990 to 2021, the number of deaths globally due to T2DM-ADR increased significantly from 164,060 (95% UI: 30,935–265,372) in 1990 to 381,416 (95% UI: 74,328–620,914) in 2021. However, the age-standardized death rate (ASDR) decreased negligibly, from 4.55 (95% UI: 0.86–7.35) per 100,000 in 1990 to 4.52 (95% UI: 0.88–7.36) per 100,000 in 2021 (**Figures 2A, B**, **Table 2**).

**Table 2:** Deaths of T2DM Between 1990 and 2021 at the Global and Country-Level.

Location	1990 DALYs (95% UI)	1990 ASR (95%UI)	2021 DALYs (95% UI)	2021 ASR (95%UI)	EAPC_CI
Global	164,060 (30,935-265,372)	4.55 (0.86-7.35)	381,416 (74,328-620,914)	4.52 (0.88-7.36)	0.61 (0.29-0.93)
United States of America	17,627 (3,785-27,385)	5.42 (1.17-8.41)	28,346 (7,190-43,601)	4.78 (1.22-7.32)	-0.58 (-1.20- -0.03)
India	15,576 (3,270-26,787)	4.03 (0.83-6.98)	58,566 (13,380-96,641)	5.53 (1.26-9.14)	2.01 (1.19-2.84)
People’s Republic of China	12,614 (1,477-22,318)	1.75 (0.21-3.10)	37,437 (4,278-67,627)	1.85 (0.21-3.34)	1.63 (1.22-2.04)
Niue	0 (0-1)	19.79 (2.8-35.09)	1 (0-1)	27.22 (5.15-49.12)	1.35 (1.06-1.64)
Tokelau	0 (0-0)	19.73 (2.49-35.55)	0 (0-1)	19.66 (3.11-34.8)	0.46 (0.25-0.66)
Fiji	187 (23-322)	56.55 (6.73-96.87)	484 (74-843)	69.84 (10.94-121.19)	1.46 (0.62-2.31)
Japan	3,150 (732-5,055)	1.9 (0.44-3.04)	2,644 (662-4,290)	0.62 (0.15-0.99)	-1.97 (-3.11- -0.81)
Singapore	63 (8-112)	3.06 (0.42-5.49)	36 (6-61)	0.43 (0.07-0.74)	-5.08 (-5.85- -4.3)

The global distribution of deaths from T2DM-ADR is increasing but varies. The three countries with the highest death tolls in 1990 and 2021 were the United States, India and China. While the United States had the highest death toll in 1990, India ranked first in 2021. The United States’ death toll increased from 17,627 (95% UI: 3,785–27,385) in 1990 to 28,346 (95% UI: 7,190–43,601) in 2021; its EAPC was -0.58. China’s death toll increased from 12,614 (95% UI: 1,477–22,318) to 37,437 (95% UI: 4,278–67,627), with an EAPC of 1.63. Moreover, the number of deaths in India increased from 15,576 (95% UI: 3,270–26,787) to 58,566 (95% UI: 13,380–96,641), with an EAPC of 2.01. Tokelau had the lowest number of deaths in both 1990 and 2021, with 0 (95% UI: 0–0) and 0 (95% UI: 0–1), respectively, and an EAPC of 0.46 (**Figures 2C, D**, **Table 2**).

However, in terms of the ASDR, Fiji recorded the highest rates globally in both 1990 and 2021, with an increase from 56.55 (95% UI: 6.73–96.87) to 69.84 (95% UI: 10.94–121.19) per 100,000 people. In contrast, in 2021, Japan and Singapore recorded the lowest rates, at 0.62 (95% UI: 0.15–0.99) and 0.43 (95% UI: 0.07–0.74) per 100,000 population, respectively (**Figure 2**, **Table 2**).

### 3.3 Global analysis of T2DM-ADR across different SDI levels

**Figure 3:**
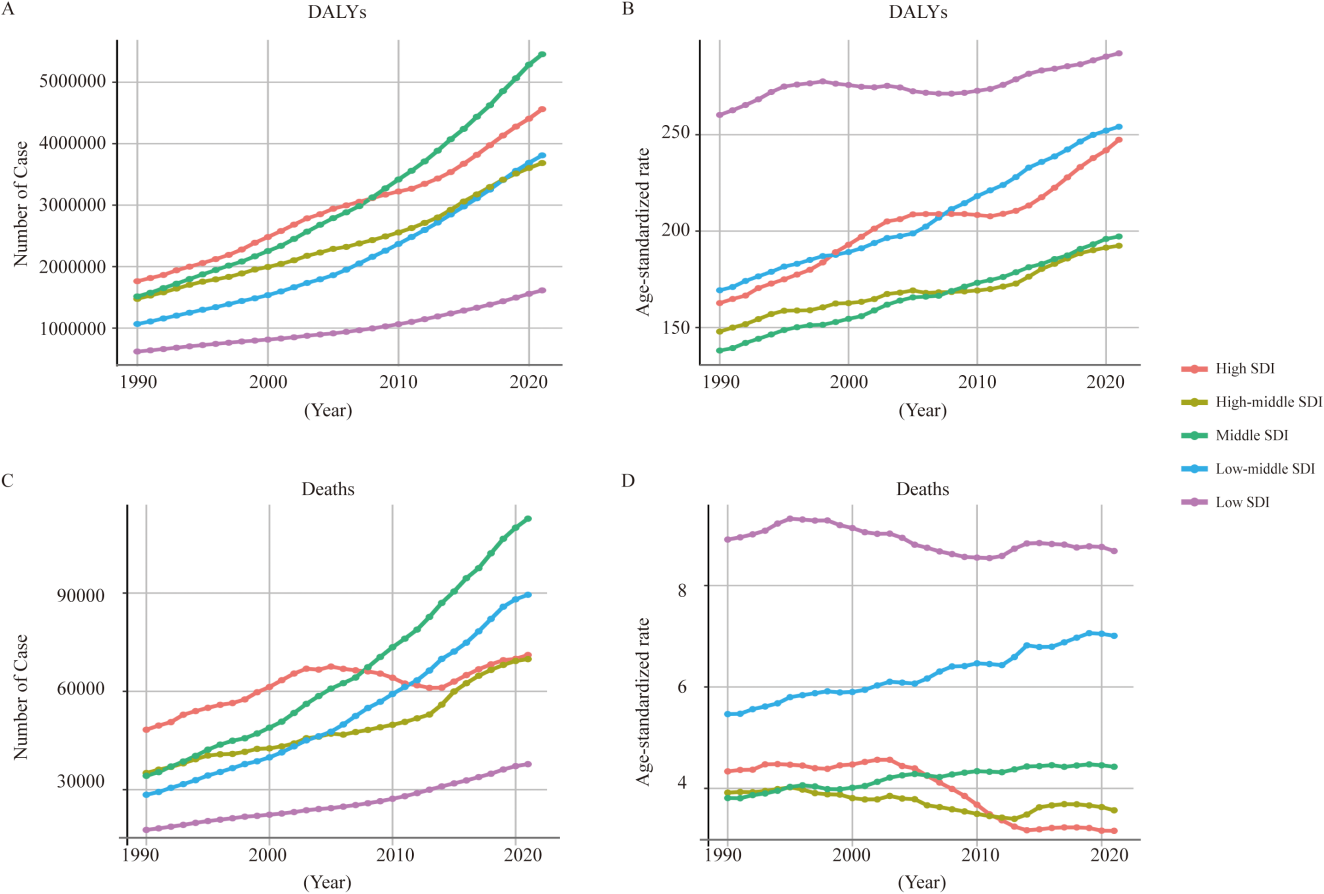
Global analysis of T2DM attributable to dietary risks across different SDI levels from 1990 to 2021. **(A)** Number of DALYs according to T2DM-ADR across areas with different SDI levels. **(B)** Age-standardized rates of DALYs in areas with different SDI levels. **(C)** Number of deaths associated with T2DM-ADR in areas with different SDI levels. **(B)** Age-standardized rates of death in areas with different SDI levels.

**Figure 4:**
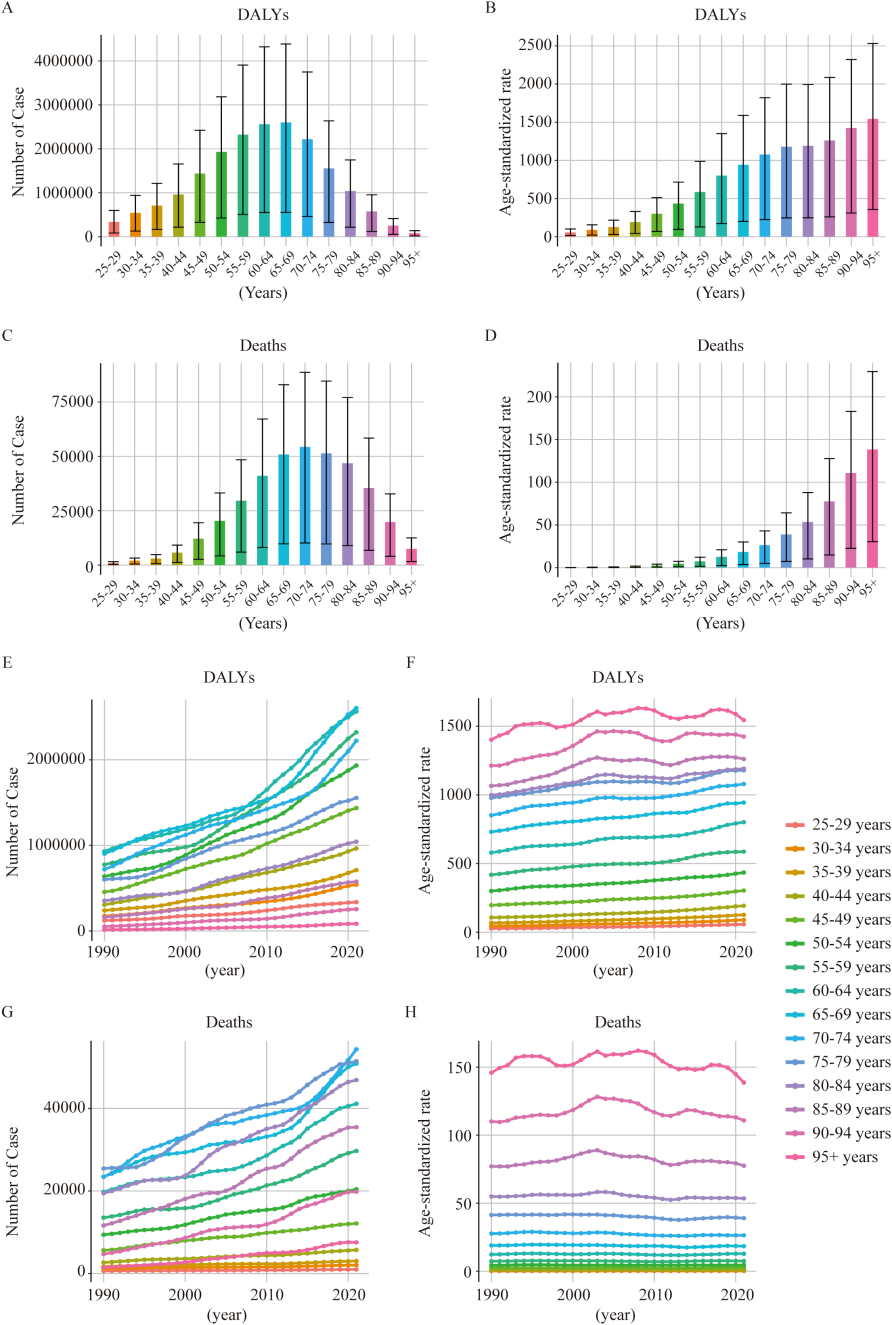
Impact of age on the global burden of T2DM attributable to dietary risks. **(A, B)** Number and age-standardized rates of DALYs in T2DM-ADR in different age groups in 2021. **(C, D)** Number and age-standardized rates of death among T2DM-ADR patients in different age groups in 2021. **(E, F)** Number and age-standardized rates of DALYs in T2DM-ADR in different age groups from 1990 to 2021. **(G, H)** Number and age-standardized rates of deaths in T2DM-ADR in different age groups from 1990 to 2021.

Epidemiological research has shown that the incidence of T2DM varies with SDI¹³. Therefore, we analyzed global trends in the burden of T2DM-ADR across different SDI levels: low, lower-middle, middle, upper-middle and high. **Figures 3**, **4** and **Tables 3**, **4** present global burden data for T2DM-ADR, including DALYs, number of deaths, YLDs and YLLs, stratified by SDI from 1990 to 2021.

**Table 3:** Global burden of T2DM attributable to dietary risks across various SDI levels.

SDI	High SDI	High-middle SDI	Middle SDI	Low-middle SDI	Low SDI
DALYs					
1990 DALYs (95%UI)	1,762,948 (387,134-2,838,065)	1,475,207 (324,314-2,399,511)	1,515,583 (264,009-2,607,534)	1,066,910 (195,040-1,811,700)	618,717 (91,674-1,037,127)
1990 ASR (95%UI)	162.69 (35.89-261.87)	147.92 (32.67-240.25)	138.06 (23.96-237.56)	169.33 (30.76-288.55)	260.26 (37.77-436.80)
2021 DALYs (95%UI)	4,561,220 (1,135,855-7,539,088)	3,685,088 (802,268-6,213,404)	5,454,307 (1,096,697-9,350,533)	3,809,852 (855,743-6,298,732)	1,613,831 (283,462-2,685,994)
2021 ASR (95%UI)	247.38 (62.69-408.23)	192.42 (41.84-324.99)	197.22 (39.52-337.88)	254.14 (55.93-422.80)	292.19 (50.31-492.72)
EAPC_CI	0.74 (0.67-0.80)	1.19 (1.09-1.30)	1.34 (1.30-1.39)	0.23 (0.17-0.29)	1.12 (1.10-1.15)
Deaths					
1990 Deaths (95%UI)	48,299 (9,767-76,055)	35,079 (7,481-55,027)	34,231 (5,801-58,053)	28,451 (4,844-47,898)	17,725 (2,409-29,621)
1990 ASR (95%UI)	4.34 (0.88-6.82)	3.92 (0.85-6.15)	3.81 (0.65-6.49)	5.47 (0.91-9.28)	8.91 (1.20-14.89)
2021 Deaths (95%UI)	71,110 (16,004-111,740)	69,853 (14,480-112,417)	112,706 (20,139-190,402)	89,481 (17,366-146,054)	37,785 (5,837-63,034)
2021 ASR (95%UI)	3.16 (0.72-4.94)	3.57 (0.74-5.74)	4.43 (0.78-7.49)	7.01 (1.34-11.53)	8.68 (1.33-14.53)
EAPC_CI	-1.39 (-1.64- -1.14)	-0.41 (-0.52- -0.3)	0.53 (0.48-0.58)	0.83 (0.79-0.87)	-0.2 (-0.27- -0.13)
YLDs					
1990 YLDs (95%UI)	799,381 (182,844-1,385,997)	715,384 (155,615-1,242,045)	633,688 (114,579-1,177,332)	352,971 (66,074-633,806)	14,2302 (22,714-254,641)
1990 ASR (95%UI)	74.85 (17.18-129.50)	69.93 (15.27-121.16)	54.39 (9.68-101.21)	52.20 (9.58-94.85)	56.73 (9.09-102.79)
2021 YLDs (95%UI)	3,240,039 (805,371-5,567,299)	2,336,437 (498,263-4,050,218)	2,801,945 (575,302-5,036,205)	170,6395 (397,521-3,019,290)	638,199 (110,126-1,136,406)
2021 ASR (95%UI)	180.96 (45.96-307.79)	124.16 (26.44-216.17)	100.06 (20.53-180.15)	107.45 (24.94-191.51)	104.84 (17.62-189.03)
EAPC_CI	2.91 (2.83-3.00)	1.83 (1.77-1.89)	1.95 (1.94-1.97)	2.38 (2.30-2.46)	1.92 (1.89-1.95)
YLLs					
1990 YLLs (95%UI)	963,567 (196,172-1,509,512)	759,823 (160,402-1,191,957)	881,895 (151,406-1,490,314)	713,939 (124,536-1,196,122)	476,415 (65,586-796,558)
1990 ASR (95%UI)	87.85 (17.96-137.51)	77.99 (16.58-122.45)	83.66 (14.26-141.55)	117.12 (20.07-196.94)	203.53 (27.77-339.88)
2021 YLLs (95%UI)	1,321,181 (308,456-2,048,555)	1,348,651 (278,787-2,165,404)	2,652,363 (490,188-4,437,487)	2,103,458 (416,811-3,406,099)	975,632 (152,301-1,606,703)
2021 ASR (95%UI)	66.43 (15.85-102.21)	68.25 (14.14-109.48)	97.16 (17.84-162.90)	146.69 (28.74-238.51)	187.36 (28.98-312.63)
EAPC_CI	-1.29 (-1.52- -1.06)	-0.64 (-0.77- -0.50)	0.45 (0.41-0.49)	0.76 (0.73-0.79)	-0.43 (-0.50- -0.36)

**Table 4:** Gender differences in the global impact of dietary risks on T2DM distribution.

Index	Sex	1990 Number (95% UI)	1990 ASR (95% UI)	2021 Number (95% UI)	2021 ASR (95% UI)	EAPC_CI
DALYs	Both	6,450,217 (1,256,122-10,613,945)	159.96 (31.17-262.82)	19,146,810 (4,147,239-31,937,618)	221.34 (47.97-368.92)	0.96 (0.92-1.01)
	Male	3,134,601 (615,407-5,196,822)	165.89 (32.56-274.73)	9,750,524 (2,078,052-16,367,491)	236.94 (50.42-397.97)	1.07 (1.03-1.12)
	Female	3,315,616 (640,715-5,432,411)	154.61 (29.91-253.10)	9,396,286 (2,069,187-15,612,534)	207.18 (45.75-344.06)	0.85 (0.8-0.9)
Deaths	Both	164,060 (30,935-265,372)	4.55 (0.86-7.35)	381,416 (74,328-620,914)	4.52 (0.88-7.36)	-0.15 (-0.22- -0.07)
	Male	73,567 (14,152-120,130)	4.65 (0.89-7.55)	183,717 (34,692-301,286)	4.85 (0.92-7.95)	0.02 (-0.06- 0.11)
	Female	90,493 (16,783-146,848)	4.47 (0.83-7.26)	197,699 (39,636-324,534)	4.25 (0.85-6.98)	-0.3 (-0.38- -0.23)
YLDs	Both	2,647,950 (553,253-4,678,966)	63.55 (13.29-112.56)	10,734,857 (2,435,669-18,705,254)	124.01 (28.15-216.28)	2.17 (2.13-2.21)
	Male	1,324,050 (271,514-2,352,382)	66.82 (13.71-118.84)	5,493,441 (1,231,653-9,641,496)	132.08 (29.59-232.38)	2.2 (2.16-2.24)
	Female	1,323,900 (281,653-2,337,455)	60.65 (12.90-107.06)	5,241,416 (1,204,016-9,089,462)	116.62 (26.84-202.35)	2.13 (2.09-2.16)
YLLs	Both	3,802,267 (718,525-6,152,372)	96.41 (18.22-155.98)	8,411,953 (1,660,756-13,664,004)	97.33 (19.23-158.11)	-0.13 (-0.2- -0.05)
	Male	1,810,551 (350,727-2,965,802)	99.08 (19.15-61.81)	4,257,083 (812,868-6,934,624)	104.86 (20.01-171.09)	0.05 (-0.02- -0.12)
	Female	1,991,716 (367,798-3,226,579)	93.96 (17.38-52.19)	4,154,870 (847,888-6,782,492)	90.56 (18.53-47.81)	-0.3 (-0.39- -0.22)

#### 3.3.1 SDI-stratified characteristics of global DALY and age-standardized DALY rates

Throughout the period from 1990 to 2021, the burden of T2DM-ADR increased across all the SDI levels. The greatest increase occurred in the middle-SDI region (260% increase from 618,717 [95% UI: 991,674 to 1,037,127] to 1,613,831 [95% UI: 283,462 to 2,685,994]), with an EAPC of 1.34. There was a 257% increase in the lower-middle-SDI area from 1,066,910 (95% UI 195, 040–1,811,700) to 3,809,852 (95% UI 855,743–6,298,732), but the EAPC was lower, 0.23. Despite having a higher base number, the higher-SDI region increased by 159%, from 1.76 million (95% UI: 387,133-2,838,065) to 4.56 million (95% UI: 1,135,855-7,539,088), which is an increase of 0.74 EAPC (see **Figures 3A, B**, **Table 3**). In 1990, the low-SDI region was affected mainly by YLDs, and the other regions (i.e., the SDI region) were affected mainly by YLLs (**Table 3**). In 2021, the high-SDI region and middle-SDI region were affected primarily by YLLs, whereas the other SDI regions were affected predominantly by YLDs (**Table 3**).

The ASR of DALYs exhibited a similar trend, with the low-SDI region demonstrating the highest ASR in both 1990 and 2021, which increased from 260.26 (95% UI: 37.77–436.80) to 292.19 (95% UI: 50.31–492.72). Concurrently, the high-SDI region exhibited a 52% increase in ASR, increasing from 162.69 (95% UI: 35.89–261.87) to 247.38 (95% UI: 62.69–408.23) (**Figures 3A, B**, **Table 3**).

#### 3.3.2 Geographical disparities in the number of deaths and ASDR

The middle-SDI region experienced the most substantial increase in deaths, which increased by 229% from 34,231 (95% UI: 5,801–58,053) to 112,706 (95% UI: 20,139–190,402), with an EAPC of 0.53. The lowest ASR was recorded in the SDI region, which reached 8.68 (95% UI: 1.33–14.53) in 2021. The death ASR of the high-SDI region decreased from 4.34 (95% UI: 0.88–6.82) to 3.16 (95% UI: 0.72–4.94), with an EAPC of -1.39. The ASR in the lower-middle-SDI region substantially increased, from 5.47 (95% UI: 0.91–9.28) to 7.01 (95% UI: 1.34–11.53). This increase was accompanied by an elevated EAPC of 0.83, as illustrated in **Figures 3C, D** and substantiated in **Table 3**.

### 3.4 Impact of age and sex on the global burden of T2DM-ADR

These results also demonstrate the effect of age on the global burden of T2DM-ADR (**Figure 4**). In 1990, the highest number of DALYs of 930,269 (95% UI 178,923 to 1,544,783) was noted in the 60–64 year age group, primarily due to YLLs. By 2021, the highest number of DALYs of 2,603,520 (95% UI: 553,323–4,386,272) was noted within the 65–69 age group. However, the age group over 95 years had the smallest numbers in both 1990 and 2021: 14,270 (95% UI: 3,061–23,201) and 84,178 (95% UI: 19,467–137,929), respectively. The increase in the number of deaths in the two age groups considered in these authors’ calculations is due to the increase in YLLs (**Figures 4A, B, E, F**).

With respect to mortality, the highest numbers of fatalities were found in the category aged 75–79 years in 1990 and the category aged 70–74 years in 2021. The number of deaths in the 25–29 year age group was the lowest in 1990 and 2021. These data show variations in the number of deaths for each age group between 1990 and 2021, with the numbers of deaths for the 25- to 29-year-old group varying over time (**Figures 4C, D, G, H**; **Supplementary Figure 1**).

**Figure 5:**
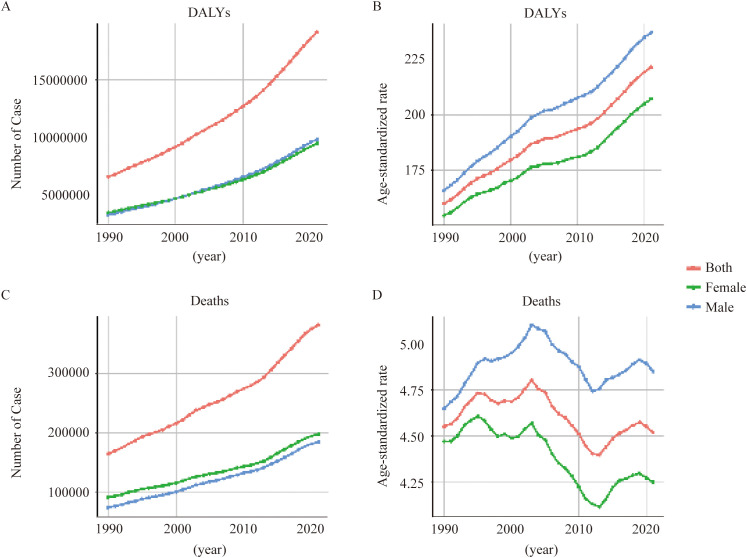
Effects of gender on the global burden of T2DM attributable to dietary risks. **(A, B)** Number and age-standardized rates of DALYs in T2DM-ADR in different sex groups from 1990 to 2021. **(C, D)** Number and age-standardized rates of deaths in T2DM-ADR in different sex groups from 1990 to 2021.

Analyses were also performed to determine sex effects on DALYs due to T2DM-ADR. When gender is accounted for, an overall increasing trend was present. YLLs contributed more in 1990, whereas YLDs began contributing more in 2021. In 1990, the number of DALYs for females was greater than that for males (3,315,616 vs. 3,134,601). However, in 2021, males dominated (9,750,524 vs. 9,396,286). We found higher age-standardized DALYs among males than among females. When we compared the mortality numbers, female deaths were greater both in 1990 and in 2021, whereas age-standardized death rates for men increased over time from 4.65 (95% UI: 0.89–7.55) to 4.85 (95% UI: 0.92–7.95). Gender differences significantly influenced the distribution of the disease burden of T2DM-ADR, with males accounting for the majority of the incremental burden (**Figure 5**, **Table 4**).

**Figure 6:**
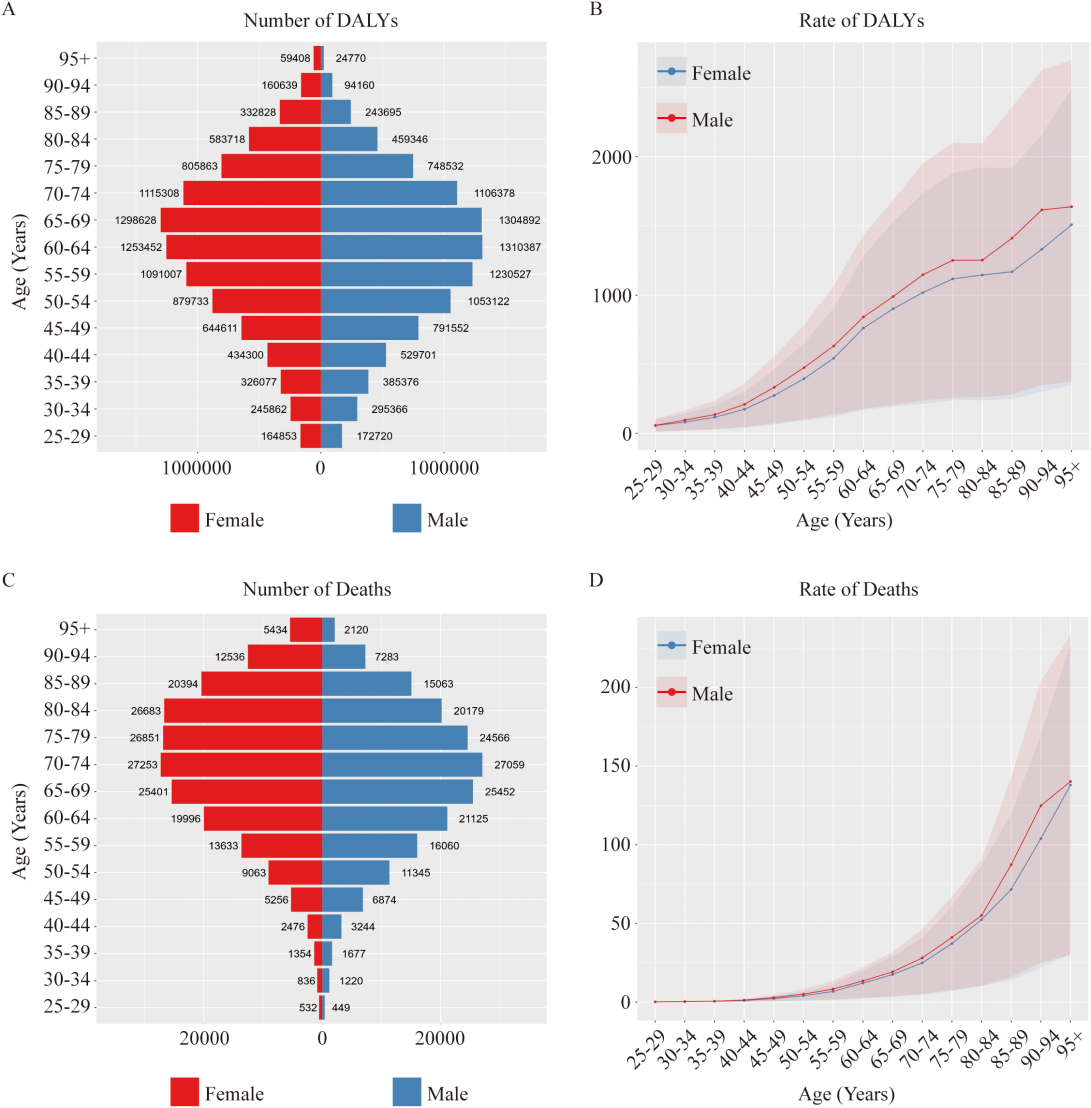
Effects of gender and age on the global burden of T2DM attributable to dietary risks. **(A, B)** Number and age-standardized rates of DALYs among T2DM-ADR patients in 2021. **(C, D)** Number and age-standardized rates of deaths among T2DM-ADRs in 2021.

On the basis of an exhaustive appraisal of age and sex distributions, the group aged 65–69 years had the greatest number of DALYs, with the number of DALYs being greater in males than in females. The 70- to 74-year-old age group had the highest mortality, and females in this age group had a higher mortality rate than the corresponding males. In terms of the data, compared with women, men had considerably higher age-standardized DALYs, deaths, YLDs and YLLs (**Figures 4**, **6**, **Supplementary Figure 2**).

### 3.5 Global burden of T2DM-ADR by GBD region

**Figure 7:**
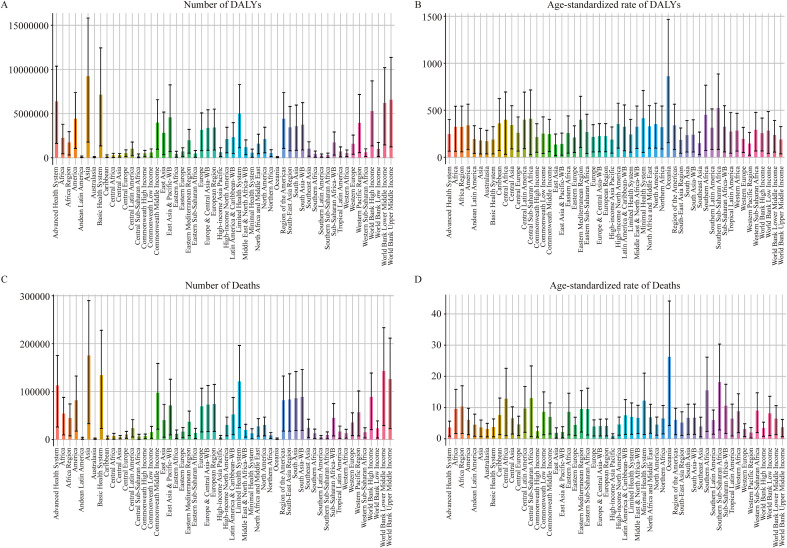
Global burden of T2DM attributable to dietary risks by GBD region. **(A, B)** Number and age-standardized rates of DALYs in T2DM-ADR in different regions in 2021. **(C, D)** Number and age-standardized rates of deaths in T2DM-ADR in different regions in 2021.

At the regional level, the burden of T2DM-ADR in all 21 GBD regions increased from 1990 to 2021; however, the global distribution of DALYs due to T2DM-ADR varied, with Asia being particularly severe, showing an increase from 2,616,083 (95% UI: 477,634–4,468,832) in 1990 to 9,250,507 (95% UI: 1,785,180–15,808,286) in 2021, a 254% increase, with an EAPC of 2.03. Oceania had the lowest DALYs in both 1990 and 2021, with 24,390 (95% UI: 3,995–41,680) and 73,953 (95% UI: 13,438–124,792), respectively (**Figure 7**, **Table 5**, **Supplementary Figure 3**, **Supplementary Tables 3**, **4**).

**Table 5:** Global burden in DALYs distribution due to dietary risk-induced T2DM across regions.

Location	1990 DALYs (95% UI)	1990 ASR (95% UI)	2021 DALYs (95% UI)	2021 ASR (95% UI)	EAPC_CI
Global	6,450,217 (1,256,122-10,613,945)	159.96 (31.17-262.82)	19,146,810 (4,147,239-31,937,618)	221.34 (47.97-368.92)	1.59 (1.37-1.81)
Advanced Health System	2,683,405 (620,596-4,281,742)	166.35 (38.64-265.16)	6,363,945 (1,632,591-10,369,271)	247.55 (64.44-401.37)	1.65 (1.14-2.17)
Asia	2,616,083 (477,634-4,468,832)	123.05 (22.49-210.03)	9,250,507 (1,785,180-15,808,286)	179.15 (34.60-305.89)	2.03 (1.72-2.34)
World Bank High Income	2,259,818 (513,137-3,612,101)	178.79 (40.81-285.68)	5,305,431 (1,347,879-8,698,552)	255.43 (66.12-417.07)	1.54 (0.99-2.08)
Europe	1,620,459 (382,412-2,569,792)	158.63 (37.64-251.34)	3,158,446 (805,892-5,084,629)	216.49 (55.87-346.31)	1.39 (0.83-1.94)
America	1,459,950 (304,313-2,388,364)	238.44 (49.57-389.89)	4,424,056 (1,056,703-7,382,646)	338.43 (81.24-564.04)	1.62 (1.43-1.8)
Basic Health System	2,059,399 (337,459-3,546,040)	131.69 (21.35-225.52)	7,167,502 (1,343,390-12,431,301)	187.56 (35.14-324.75)	2.12 (1.83-2.41)
World bank upper-middle income	2,045,619 (370,644-3,490,999)	130.30 (23.57-222.16)	6,580,176 (1,240,051-11,351,849)	188.65 (35.64-326.01)	2.05 (1.81-2.29)
Australasia	32,163 (6,003-52,803)	138.34 (25.94-226.59)	85,146 (17,702-143,240)	169.44 (35.79-286.11)	1.06 (0.6-1.53)
Andean Latin America	29,000 (6,298-49,770)	134.57 (28.30-232.81)	116,689 (25,094-201,584)	192.80 (41.00-334.98)	1.95 (1.45-2.45)
Oceania	24,390 (3,995-41,680)	742.10 (117.64-1266.63)	73,953 (13,438-124,792)	862.05 (154.31-1465.64)	0.83 (-0.01-1.69)
East Asian	813,257 (104,457-1,477,201)	86.82 (11.17-156.93)	2,835,683 (357,683-5,172,916)	134.85 (17.33-247.24)	2.44 (2.15-2.73)

However, with respect to age-standardized DALYs, Oceania had the highest values in both 1990 (742.10 [95% UI: 117.64–1266.63]) and 2021 (862.05 [95% UI: 154.31–1465.64]), with an EAPC of 0.83. The minimum values were documented in East Asia, with 86.82 (95% UI: 11.17–156.93) and 134.85 (95% UI: 17.33–247.24), respectively, and an EAPC of 2.44 (**Figures 7A, B**, **Table 5**).

With respect to mortality, Asia has consistently led globally, with an increase from 57,953 deaths (95% UI: 10,386–97,935) in 1990 to 175,271 deaths (95% UI: 33,147–289,931) in 2021, with an EAPC of 1.32. Oceania continued to demonstrate the lowest number of deaths, with 686 deaths (95% UI: 109–1,194) in 1990 and 1,788 deaths (95% UI: 287–3,031) in 2021, yielding an EAPC of 0.36. According to the most recent data, Oceania consistently exhibited the highest age-standardized mortality rates in both 1990 and 2021, with 25.9 cases per 100,000 (95% UI: 4.11–44.44) and 26.27 cases per 100,000 (95% UI: 4.25–44.14), respectively. In 1990, Eastern Europe recorded the lowest rate at 1.58 (95% UI: 0.33–2.4) per 100,000. By 2021, the high-income Asia Pacific region had the lowest rate of 0.99 (95% UI: 0.2–1.61) per 100,000, accompanied by an EAPC of -1.19 (**Figures 7C, D**, **Table 6**).

**Table 6:** Global trends in deaths due to dietary risk-induced T2DM across regions.

Location	1990 DALYs (95% UI)	1990 ASR (95% UI)	2021 DALYs (95% UI)	2021 ASR (95% UI)	EAPC_CI
Global	164,060 (30,935-265,372)	4.55 (0.86-7.35)	381,416 (74,328-620,914)	4.52 (0.88-7.36)	0.61 (0.29-0.93)
Advanced Health System	72,002 (15,446-111,809)	4.42 (0.95-6.87)	112,591 (25,987-175,303)	3.66 (0.85-5.65)	-0.14 (-0.78-0.5)
World Bank High Income	63,906 (13,387-99,570)	4.89 (1.03-7.61)	88,870 (20,325-138,668)	3.44 (0.8-5.33)	-0.61 (-1.32-0.1)
Asia	57,953 (10,386-97,935)	3.31 (0.59-5.58)	175,271 (33,147-289,931)	3.62 (0.68-6)	1.32 (0.83-1.81)
Europe	45,616 (9,978-70,479)	4.45 (0.98-6.88)	69,223 (15,741-106,813)	3.94 (0.6-6.05)	0.27 (-0.41-0.96)
America	38,533 (7,504-61,638)	6.38 (1.24-10.21)	81,938 (17,521-132,410)	6.05 (1.3-9.78)	0.17 (-0.07-0.40)
Australasia	935 (165-1,508)	4.07 (0.72-6.56)	1,861 (362-2,945)	3.15 (0.61-4.94)	-0.34 (-0.96-0.27)
Andean Latin America	769 (151-1,295)	3.93 (0.75-6.63)	2,611 (479-4,588)	4.51 (0.82-7.91)	1.34 (0.75-1.94)
Oceania	686 (109-1,194)	25.9 (4.11-44.44)	1,788 (287-3,031)	26.27 (4.25-44.14)	0.36 (-0.77-1.5)
World Bank Lower Middle Income	44,489 (8,095-74,752)	4.96 (0.89-8.40)	142,845 (29,108-233,417)	6.47 (1.31-10.62)	1.47 (0.61-2.33)
Basic Health System	45,224 (7,087-76,842)	3.49 (0.55-5.95)	134,193 (22,846-227,963)	3.74 (0.63-6.36)	1.44 (0.99-1.9)
Eastern Europe	4,460 (929-6,784)	1.58 (0.33-2.4)	16,211 (3,665-25,028)	4.47 (1.01-6.9)	2.68 (1.56-3.82)
High-income Asia Pacific	4,308 (938-7,000)	2.21 (0.48-3.6)	5,092 (1,063-8,444)	0.99 (0.2-1.61)	-1.19 (-2.05- -0.32)

### 3.6 Prediction of global trends of T2DM-ADR from 2030 to 2050

**Figure 8:**
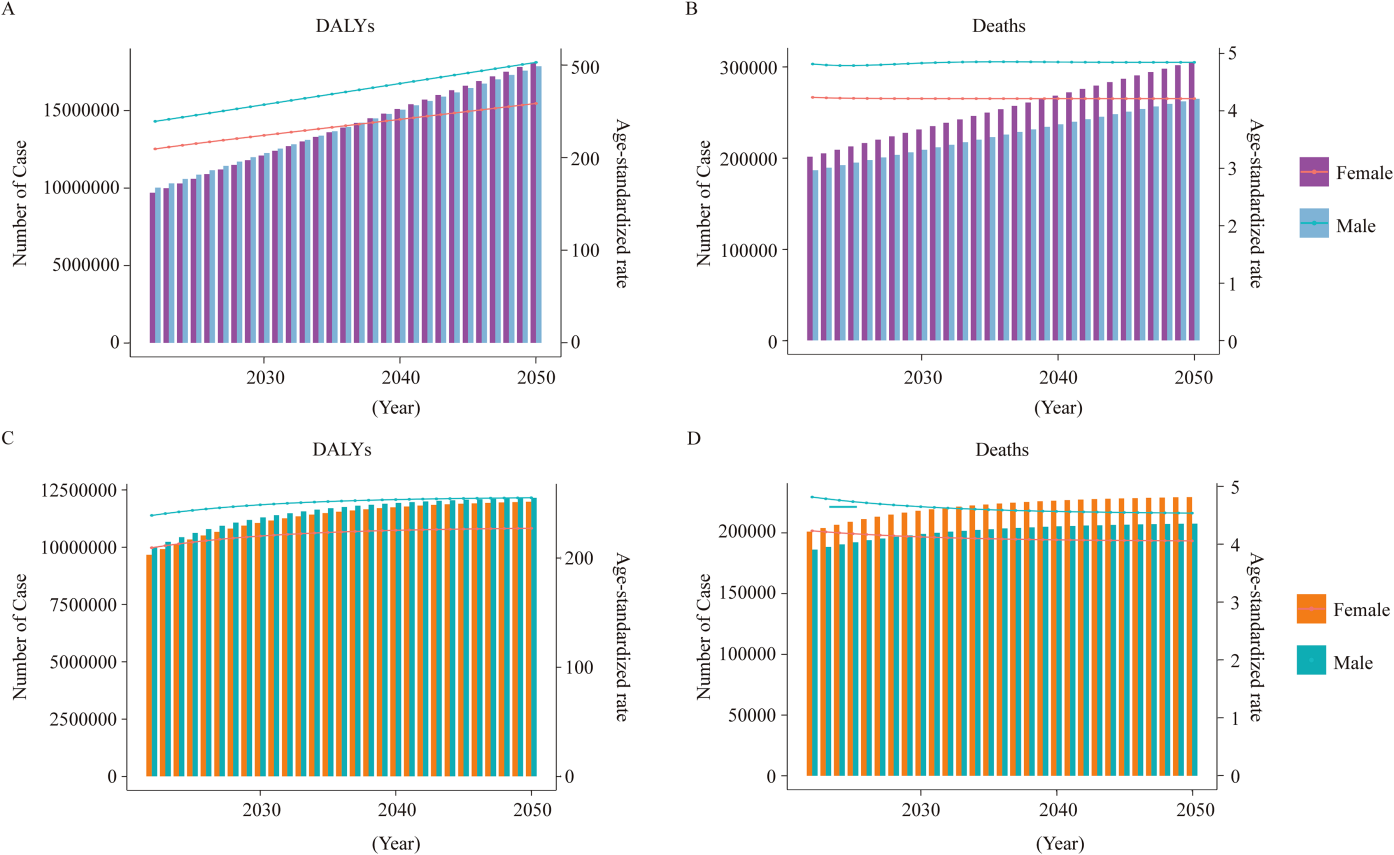
Prediction of global trends of T2DM attributable to dietary risks from 2030 to 2050. **(A, B)** Prediction of the number of DALYs and deaths in terms of T2DM-ADR by the ARIMA model in different sex groups for 2030 to 2050. **(C, D)** Prediction of the number of DALYs and deaths in terms of T2DM-ADR by the ES model in different sex groups from 2030 to 2050.

Considering the aforementioned results, we used the ARIMA and ES models to forecast the impact of dietary risk on the impending T2DM-ADR burden from 2030 to 2050. This model predicts a global increase in the prevalence of T2DM-ADR. By 2050, it is projected that the global burden of T2DM-ADR in males will total 17,865,944 DALYs (95% UI: 14,123,935–21,607,953), while in females, it will amount to 18,121,264 DALYs (95% UI: 14,253,245–21,989,282). YLDs are expected to be the primary contributor to this burden. The projected death toll for males is estimated to be 264,822 (95% UI: 115,656–413,989), and for females, it is 305,383 (95% UI: 124,195–486,570) in 2050. Additionally, both age-standardized DALYs and YLDs tended to increase (**Figure 8** and **Supplementary Figure 4**).

## 4 Discussion

Based on the GBD database, this study comprehensively analyzed the changes in the global burden of T2DM-ADR from 1990 to 2021 and predicted future trends. Globally, both DALYs and deaths attributable to T2DM-ADR increased from 1990 to 2021, underscoring the substantial role of dietary factors in T2DM-ADR.

### 4.1 Global and regional trends

Our analyses revealed notable regional heterogeneity in the global burden of T2DM-ADR, reflecting the influence of interactions among lifestyle changes, economic development, and region-specific factors on health problems. There is, for example, a dramatic increase in the burden of T2DM-ADR in Asia, particularly in China (where DALYs increased by almost 251% between 1990 and 2021), a pattern typical of the evolution of global development. This sharp rise in coping with mortality not only jeopardizes an individual’s health but also seriously threatens the health system and results in high economic and social costs in the long term. Although economic growth and the spread of unhealthy dietary patterns, including processed foods high in calories, fats and sugars, and refined carbohydrates, have gradually increased the prevalence of obesity and metabolic syndrome, leading to an increase in the burden of T2DM. This sharp rise in coping with mortality not only jeopardizes an individual’s health but also seriously threatens the health system and results in high economic and social costs in the long term (13, 14).

In contrast, the high ASR observed in small-island developing countries in Oceania, as well as the Marshall Islands, indicates a distinct public health issue. Even with a small population base, their markedly elevated ASR highlights that health risks in smaller populations cannot be ignored. This phenomenon is often related to changes in traditional dietary patterns due to global food trade, whereby locally fiber-rich and nutritionally balanced traditional foods are being replaced by high-sugar, high-fat, low-nutrient-density imported processed foods. Additionally, the rising obesity rates accompanying modernization have further contributed to the high incidence of T2DM (15, 16). The obvious distinctions between these places highlight that T2DM is a complex interaction of multiple factors, including economic development level, cultural shifts, food system changes, and geographic environment; rather than being a single pattern, the prevalence of T2DM is an outcome of complex interactions. These findings also suggest that public health strategies must be customized to local circumstances, addressing “nutrition transition” issues in developing countries while emphasizing the protection of traditional diets and the guidance of modern healthy diets for specific vulnerable groups, such as those in small island developing countries (17).

### 4.2 SDI regions and dietary risks

The global impact of different SDI dietary risks on T2DM is significantly different. The increase is greatest in the middle-SDI regions, whereas it is relatively low in the high-SDI regions. These findings indicate that there is no significant correlation between economic development level and the effect of dietary risk on T2DM burden. Moreover, although the low-SDI regions have a lower baseline burden, the ASR remains the highest and does not significantly improve. The cause is likely due to multiple reasons: 1) Traditional diets in these regions often contain high amounts of sugar, fat, and salt but lack sufficient fruits, vegetables, and whole grains (18, 19). For example, refined carbohydrates and high-sugar beverages are widely used in the diet of South Asia, while sub-Saharan Africa faces the dual challenges of malnutrition and an unhealthy diet (20, 21). 2) Lifestyle: The rapid urbanization process and westernization of lifestyles have resulted in decreased physical activity, further exacerbating the risk of T2DM (22, 23). 3) Socioeconomic factors: Residents in low- and middle-income regions may face more health risks, such as a lack of healthy food choices, insufficient medical resources, and relatively low health awareness (24, 25). The middle-SDI regions show the greatest increase in DALYs, deaths, and YLLs, indicating that health risks are caused by dietary imbalances during rapid urbanization. Although the increase in high-SDI regions is relatively small, the overall burden remains high due to their large population base. Therefore, dietary factors play a key role in the prevalence of T2DM, and their impact varies significantly by region and economic level. These findings suggest that public health policies should focus on improving dietary habits and lifestyles in low- and middle-income regions.

### 4.3 Age and sex differences

The effects of age and sex on the burden of T2DM caused by dietary risk, analyzed in this study, illustrate the complexity of this global health issue. In terms of age, people older than 60 years, especially those aged 65–69 and 70–74 years, experienced significant increases in DALYs, YLLs, and death tolls, which is highly consistent with the observed physiological patterns. As individuals age, their physical functions naturally decline, metabolism slows down, and pancreatic beta cell function may gradually diminish, accompanied by reduced insulin sensitivity (26). In addition, years of poor dietary habits and lifestyles make elderly individuals a high-risk group for T2DM and its serious complications, such as cardiovascular disease, kidney disease, and retinopathy. The body’s compensation and resistance to diabetes and its long-term consequences are weakened, leading to a concentrated outbreak of disease burden at this stage (27, 28). Moreover, sex differences exhibit dynamic changes. In 1990, compared with males, females had a greater number of DALYs, which may reflect that females generally lived longer, accumulated longer periods of illness, or the influence of specific sociocultural factors (such as metabolic changes during pregnancy and postpartum, or certain traditional dietary patterns having a greater impact on women) at that time (29, 30). However, by 2021, males exceeded females in T2DM-ADR-related DALYs, with higher age-standardized DALYs and YLLs, indicating a greater burden of T2DM in males. This finding may be related to increased male exposure to unhealthy lifestyles, including smoking, excessive alcohol consumption, high-fat and high-calorie diets, and sedentary behavior, as well as occupational stress and social role expectations (31). In addition, males may have lower levels of health awareness, disease screening, and early intervention, leading to faster disease progression and earlier occurrence of complications and thus increasing the burden of death and disability (32). This phenomenon suggests that sex-specific and age-stratified T2DM prevention and control strategies should receive attention, with targeted interventions for different populations.

### 4.4 GBD regions and DALYs

From 1990 to 2021, the burden of T2DM-ADR increased in all 21 GBD regions, but the global distribution was highly unequal. Asia indeed saw substantial increases in DALYs and death numbers, substantially greater than those in any other region because of its size and as a consequence of lifestyle changes induced by economic development. In contrast, the situation regarding age-standardized indicators was completely different. Oceania had the lowest total numbers of DALYs and deaths but ranked at the top for age-standardized DALYs and deaths each time, suggesting that having the oldest population, high-risk exposure, or characteristics of the health care system results in a somewhat heavier burden. The East Asian region shows a contradictory phenomenon: its age-standardized DALYs and mortality rates are the lowest, but those DALYs increase rapidly; the reasons include the large population base of the East Asian region, the fast ageing process, and better medical record completion. Similarly, the high-income Asia Pacific region has the smallest increase in deaths and even a negative increase in deaths, revealing that this region may benefit from good medical care and medical management of disease. On the other hand, comparisons standardized by age structure should be considered in global prevention. Moreover, specific interventions should focus on regional features such as population characteristics, economic level, and medical resources to effectively address the global threat of dietary risk to individuals with T2DM (33).

Given the uneven global distribution and regional differences mentioned above, projections of future trends further underscore the imperative of targeted interventions. According to the ARIMA model, the burden of T2DM-ADR is expected to continue increasing between 2030 and 2050, suggesting that global public health policies should pay greater attention to the role of dietary factors in preventing T2DM through comprehensive, multilevel interventions. This includes strengthening health education to promote balanced diets and reduce the intake of high-sugar, high-fat, and low-fiber foods; encouraging the food industry to produce healthier products; and increasing financial support for research on the prevention and treatment of T2DM.

### 4.5 Implications for policy and intervention

Policy interventions and the core and evidence-based strategy. For instance, taxing sugar-sweetened beverages has been shown to reduce consumption (34); establishing nutritional standards in the public sector and restricting the sale of unhealthy foods in schools can increase dietary quality and help prevent childhood obesity (35); and modifying agricultural subsidies may improve access to and affordability of healthy foods (36). Additionally, popularizing health knowledge and promoting multisectoral collaboration are essential.

Technological innovation and digital health tools offer new possibilities for personalized prevention. Mobile health applications, wearable devices, and intelligent nutritional guidance can provide real-time feedback and behavioral nudges, improving the adherence to and effectiveness of interventions. Studies indicate that these tools lead to significantly better outcomes in terms of weight loss and blood glucose improvement than conventional care does (37). Overall, implementing these integrated measures could help alleviate the increasing trend of T2DM.

### 4.6 Study limitations

Although this study used comprehensive GBD data, several limitations still exist. First, T2DM onset and development result from the combined effect of multiple risk factors. This study primarily assessed the disease burden attributable to dietary risk, which does not imply that diet is the only determinant. We acknowledge that other important modifiable factors (such as physical activity levels), nonmodifiable factors (such as genetic susceptibility), and macrosocial environmental factors (such as urbanization processes and accompanying lifestyle changes) are closely related to T2DM risk and may interact with dietary risk. Second, diet risk assessment based on dietary surveys may inherently be subject to reporting bias and measurement error. Third, our predictive models used United Nations population projections to calibrate for prospective demographic changes. Since these projections carry inherent uncertainty, significant deviations from anticipated trends in any region could affect the accuracy of our burden estimates, notably for crude metrics such as DALYs. Future studies that integrate multidimensional data (such as genetics, exercise, and environmental exposure) will contribute to a more comprehensive understanding of the etiologic network of T2DM and provide a basis for the development of more targeted comprehensive prevention and control strategies.

### 4.7 Future directions

Future studies on T2DM and dietary risk should concentrate on two complementary aspects: etiological clarification and specific intervention. Etiological priorities include elucidating diet-T2DM causal pathways between diet and T2DM at both the individual and population levels, evaluating dietary intervention effectiveness (especially in resource-limited areas), examining the combined effects of diet and other lifestyle factors, and investigating vulnerable subgroups (e.g., children and elderly individuals) for stratified prevention. Intervention strategies demand a holistic, multilevel approach: individual-level evidence-based dietary patterns and restriction of added sugars and saturated fats; community and school initiatives in nutritional education and healthy food environments; policy-level enhancements, including clearer labeling, restrictions on unhealthy food marketing, and subsidies for nutritious foods; and the integration of nutritional counseling into routine health care, fostering a multidisciplinary approach to management.

## 5 Conclusions

Analysis of the GBD database revealed the substantial impact of dietary risk factors on the global burden of T2DM. Without effective interventions, the burden of T2DM is projected to increase in the next few decades, posing a significant challenge to public health systems. Therefore, fostering multistakeholder collaboration among governments, the food industry, health care providers, and communities is crucial. This collaboration is vital for coordinated health education efforts and for increasing the effectiveness of health promotion initiatives, thereby alleviating the future burden of T2DM.

## Data Availability

The original contributions presented in the study are included in the article/**Supplementary Material**. Further inquiries can be directed to the corresponding authors.
