# Supplementary material for: A detailed examination of the worldwide impact of type 2 diabetes linked to dietary risks: insights from the Global Burden of Disease Study (1990–2021)
Source: Front Endocrinol (Lausanne). 2026 Feb 20;17:1701350. doi: 10.3389/fendo.2026.1701350 (PMC12963013; doi:10.3389/fendo.2026.1701350)

**Global Burden of Type 2 Diabetes Attributable to Dietary Risks: A Comprehensive Analysis Based on the Global Burden of Disease Study (1990-2021)**

**Supplemental Table 1. YLDs Due to T2DM from 1990 to 2021: Global and Country-Level Analysis.**

| Location | 1990  YLDs (95% UI) | 1990  ASR (95%UI) | 2021  YLDs (95% UI) | 2021  ASR (95%UI) | EAPC_CI |
| --- | --- | --- | --- | --- | --- |
| Global | 2,647,950  (553,254-4,678,966) | 63.55  (13.29-112.56) | 10,734,857  (2,435,669-18,705,254) | 124.01  (28.15-216.28) | 2.75  (2.55-2.94) |
| United States of America | 289,145  (67,401-497,556) | 96.64  (22.66-166.67) | 1,401,784  (368,020-2,384,840) | 263.75  (70.69-443.70) | 3.96  (3.52-4.4) |
| India | 226,499  (49,337-408,073) | 42.77  (9.28-77.89) | 1,059,759  (263,550-1,886,381) | 81.14  (20.10-144.32) | 2.62  (2.16-3.08) |
| People’s Republic of China | 450,700  (62,561- 863,630) | 47.20  (6.55-90.03) | 1,899,437  (243,824-3,606,789) | 95.34  (12.55-180.95) | 3.24  (2.96-3.51) |
| Niue | 3  (1-6) | 161.56  (28.47-298.12) | 7  (2-13) | 350.84  (73.29-644.35) | 2.96  (2.74-3.18) |
| Tokelau | 3  (0-5) | 189.70  (24.44-361.53) | 5  (1-9) | 347.25  (63.21-636.74) | 2.3  (2.12-2.47) |
| Fiji | 808  (113-1,510) | 187.38  (24.99-352.45) | 3,153  (600-5,734) | 368.39  (71.17-670.02) | 2.84  (2.33-3.36) |
| Trinidad and Tobago | 1,936  (513-3,447) | 220.29  (57.39-391.98) | 6,986  (1,945-12,395) | 367.71  (104.33-647.95) | 2.61  (2.34-2.89) |
| Marshall Island | 45  (5-84) | 218.92  (26.08-408.49) | 205  (29-379) | 448.79  (63.70-837.66) | 3.52  (2.72-4.33) |
| Democratic People’s Republic of Korea | 4,289  (773-7,978) | 23.54  (4.26-44.00) | 17,281  (3,427-32,577) | 51.80  (10.26-97.38) | 3.2  (3.04-3.36) |

**Supplemental Table 2. YLLs Due to T2DM from 1990 to 2021: Global and Country-Level Analysis.**

| Location | 1990  YLLs (95% UI) | 1990  ASR (95%UI) | 2021  YLLs (95% UI) | 2021  ASR (95%UI) | EAPC_CI |
| --- | --- | --- | --- | --- | --- |
| Global | 3,802,267  (718,525-6,152,372) | 96.41  (18.22-155.98) | 8,411,953  (1,660,756-13,664,004) | 97.33  (19.22-158.11) | 0.52  (0.28-0.77) |
| United States of America | 374,083  (81,248-575,918) | 121.08  (26.40-186.03) | 591,679  (154,716-899,809) | 108.16  (28.64-163.45) | -0.56  (-1.08- -0.03) |
| India | 400,043  (85,242-681,681) | 86.90  (18.29-149.17) | 1,334,169  (307,109-2,196,870) | 113.41  (26.02-187.11) | 1.66  (1.02-2.3) |
| People’s Republic of China | 319,003  (36,625- 560,213) | 37.91  (4.44-66.88) | 802,976  (92,123-1,444,696) | 37.84  (4.36-68.10) | 1.24  (0.9-1.58) |
| Niue | 10  (1-18) | 459.06  (67.88-807.19) | 13  (3-25) | 617.90  (119.49-1131.80) | 1.3  (1.01-1.6) |
| Tokelau | 6  (1-12) | 461.16  (60.09-831.78) | 7  (1-12) | 458.71  (72.90-824.28) | 0.34  (0.17-0.52) |
| Fiji | 5,452  (691-9,332) | 1377.57  (167.92-2364.23) | 13,011  (1,922-22,689) | 1610.12  (167.92-2364.23) | 1.27  (0.67-1.87) |
| Trinidad and Tobago | 6,581  (1,713-10,370) | 779.82  (202.57-1228.43) | 11,234  (2,887-19,496) | 580.28  (149.94-1004.61) | -0.33  (-0.66- -0.01) |
| Marshall Island | 115  (12-206) | 672.44  (70.28-1211.40) | 397  (59-761) | 1002.20  (137.95-1915.53) | 2.42  (1.37-3.47) |
| Democratic People’s Republic of Korea | 5,443  (842-10,080) | 32.39  (5.03-59.15) | 12,775  (2,023-23,751) | 37.93  (6.03-70.18) | 1.47  (1.26-1.68) |

**Supplemental Figure 1. The impact of age and gender on YLDs and YLLs of the global T2DM caused by dietary risks.**

**
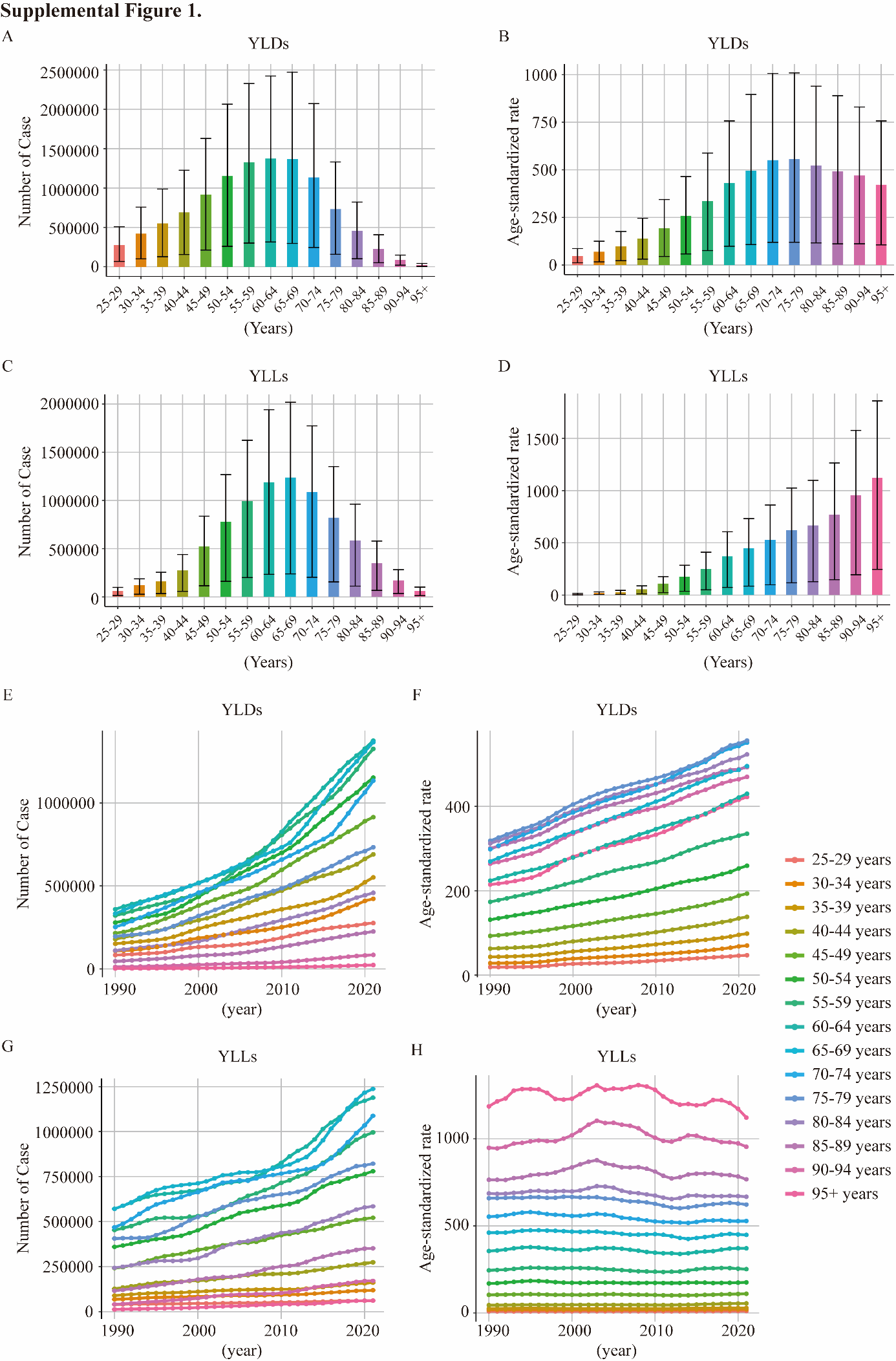
**

**Supplemental Figure 2. The comprehensive analysis of age and gender on YLDs and YLLs of the global T2DM caused by dietary risks.**

**
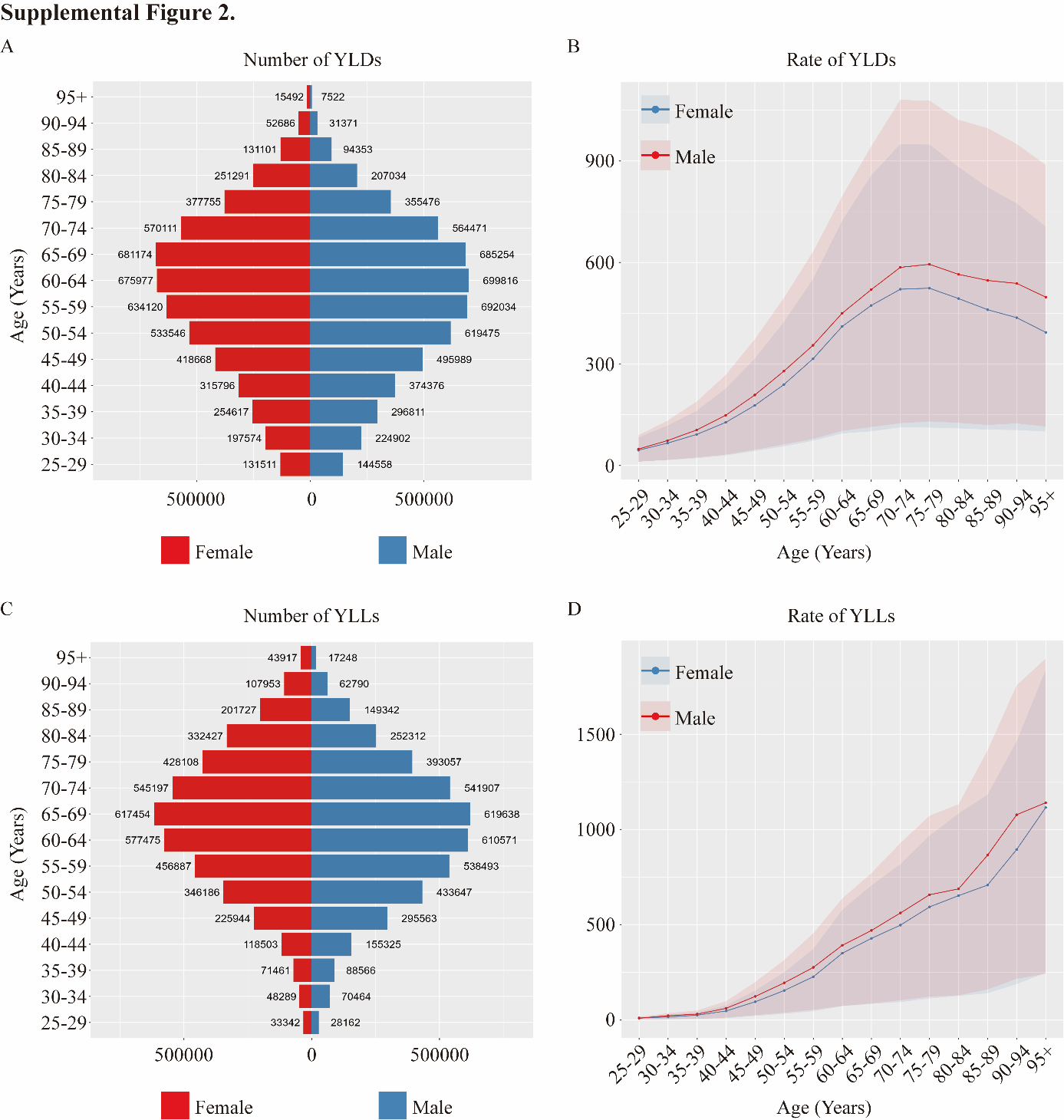
**

**Supplemental Table 3. Global Distribution of YLDs Attributable to Dietary Risk-Induced T2DM Across Regions.**

| Location | 1990  YLDs (95% UI) | 1990  ASR (95%UI) | 2021  YLDs (95% UI) | 2021  ASR (95%UI) | EAPC_CI |
| --- | --- | --- | --- | --- | --- |
| Global | 2,647,950  (553,254-4,678,966) | 63.55  (13.29-112.56) | 10,734,857  (2,435,669-18,705,254) | 124.01  (28.15-216.28) | 2.75  (2.55-2.94) |
| Advanced Health System | 1,233,659  (292,166-2,111,304) | 77.31  (18.37-132.46) | 4,303,525  (1,085,699-7,329,295) | 173.21  (44.66-292.59) | 3.12  (2.64-3.61) |
| Asia | 1,166,937  (214,478-2,135,811) | 51.8  (9.59-94.65) | 5,261,423  (1,072,621-9,396,119) | 101.1  (20.63-181.01) | 2.93  (2.67-3.19) |
| World Bank High Income | 1,003,460  (236,737-1,735,593) | 80.95  (19.18-139.98) | 3,712,272  (930,531-6,343,382) | 185.06  (47.52-314.87) | 3.2  (2.68-3.71) |
| Europe | 735,305  (176,014-1,249,395) | 73.16  (17.58-123.88) | 1,971,081  (496,943-3,326,066) | 142.63  (36.81-238.32) | 2.55  (2.04-3.06) |
| America | 569,221  (125,756-999,464) | 92.64  (20.46-162.75) | 2,586,785  (634,494-4,456,674) | 198.94  (49.14-341.68) | 3.24  (3.06-3.42) |
| Basic Health System | 909,659  (159,525-1,691,424) | 55.20  (9.62-102.91) | 4,038,832  (748,654-7,328,157) | 105.30  (19.57-191.52) | 3.03  (2.78-3.29) |
| World bank upper-middle income | 962,780  (179,283-1,761,424) | 58.95  (11-107.69) | 3,772,765  (691,369-6,813,781) | 109.13  (20.09-197.55) | 2.87  (2.65-3.10) |
| Australasia | 14,009  (2,638-24,615) | 60.37  (11.45-105.40) | 54,462  (11,398-96,623) | 112.19  (23.82-198.38) | 2.75  (2.32-3.19) |
| Andean Latin America | 9,471  (2,021-17,676) | 42.54  (8.83-79.83) | 56,082  (12,603-103,006) | 91.67  (20.26-168.45) | 3.41  (2.93-3.89) |
| Oceania | 4,216  (771-7,762) | 117.67  (21.44-217.03) | 22,630  (4,669-41,316) | 239.62  (50.13-443.51) | 2.65  (1.92-3.39) |
| East Asian | 466,036  (64,732-888,500) | 47.05  (6.53-89.39) | 1,973,668  (255,311-3,732,181) | 95.53  (12.66-180.85) | 3.25  (2.97-3.53) |

**Supplemental Table 4. Global Distribution of YLLs Attributable to Dietary Risk-Induced T2DM Across Regions.**

| Location | 1990  YLLs (95% UI) | 1990  ASR (95%UI) | 2021  YLLs (95% UI) | 2021  ASR (95%UI) | EAPC_CI |
| --- | --- | --- | --- | --- | --- |
| Global | 3,802,267  (718,525-6,152,372) | 96.41  (18.22-155.98) | 8,411,953  (1,660,756-13,664,004) | 97.33  (19.22-158.11) | 0.52  (0.28-0.77) |
| Advanced Health System | 1,449,746  (313,450-2,237,923) | 89.03  (19.33-137.47) | 2,060420  (488,917-3,161,303) | 74.34  (17.92-113.44) | -0.33  (-0.89-0.23) |
| Asia | 1,449,146  (260,077-2,433,219) | 71.25  (12.79-120.27) | 3,989,084  (765,909-6,550,475) | 78.05  (14.97-128.26) | 1.13  (0.78-1.49) |
| World Bank High Income | 1,256,358  (264,452-1,948,031) | 97.84  (20.68-151.56) | 1,593,159  (376,646-2,453,703) | 70.37  (16.99-107.62) | -0.75  (-1.36- -0.14) |
| Europe | 885,154  (195,391-1,358,206) | 85.48  (18.97-131.22) | 1,187,364  (273,666-1,814,075) | 73.85  (17.16-112.61) | -0.06  (-0.69- -0.57) |
| America | 890,727  (175,289-1,421,105) | 145.8  (28.65-232.7) | 1,837,271  (406,521-2,951,610) | 139.48  (31.01-223.85) | 0.11  (-0.1-0.33) |
| Basic Health System | 1,149,740  (182,201-1,940,831) | 76.48  (12.06-129.65) | 3,128,670  (551,467-5,262,490) | 82.26  (14.43-138.43) | 1.25  (0.92-1.57) |
| World bank upper-middle income | 1,082,839  (187,761-1,789,911) | 71.35  (12.36-118.28) | 2,807,411  (516,146-4,694,434) | 79.52  (14.63-132.81) | 1.19  (0.93-1.45) |
| Australasia | 18,154  (3,226-29,023) | 77.97  (13.97-124.31) | 30,684  (6,018-47,791) | 57.25  (11.33-88.71) | -0.81  (-1.35- -0.28) |
| Andean Latin America | 19,529  (4,087-33,007) | 92.03  (18.80-155.37) | 60,607  (12,140-106,620) | 101.13  (20.03-177.89) | 1.02  (0.51-1.54) |
| Oceania | 20,174  (3,213-35,401) | 624.43  (98.95-1084.07) | 51,323  (8,177-87,023) | 622.42  (99.85-1055.02) | 0.31  (-0.57-1.19) |
| East Asian | 347,221  (39,725-612,774) | 39.77  (4.64-70.5) | 862,014  (100,388-1,549,358) | 39.32  (4.6-70.79) | 1.12  (0.77-1.46) |

**Supplemental Figure 3. Analysis of YLDs and YLLs of T2DM Due to Dietary Risks by GBD Region**.
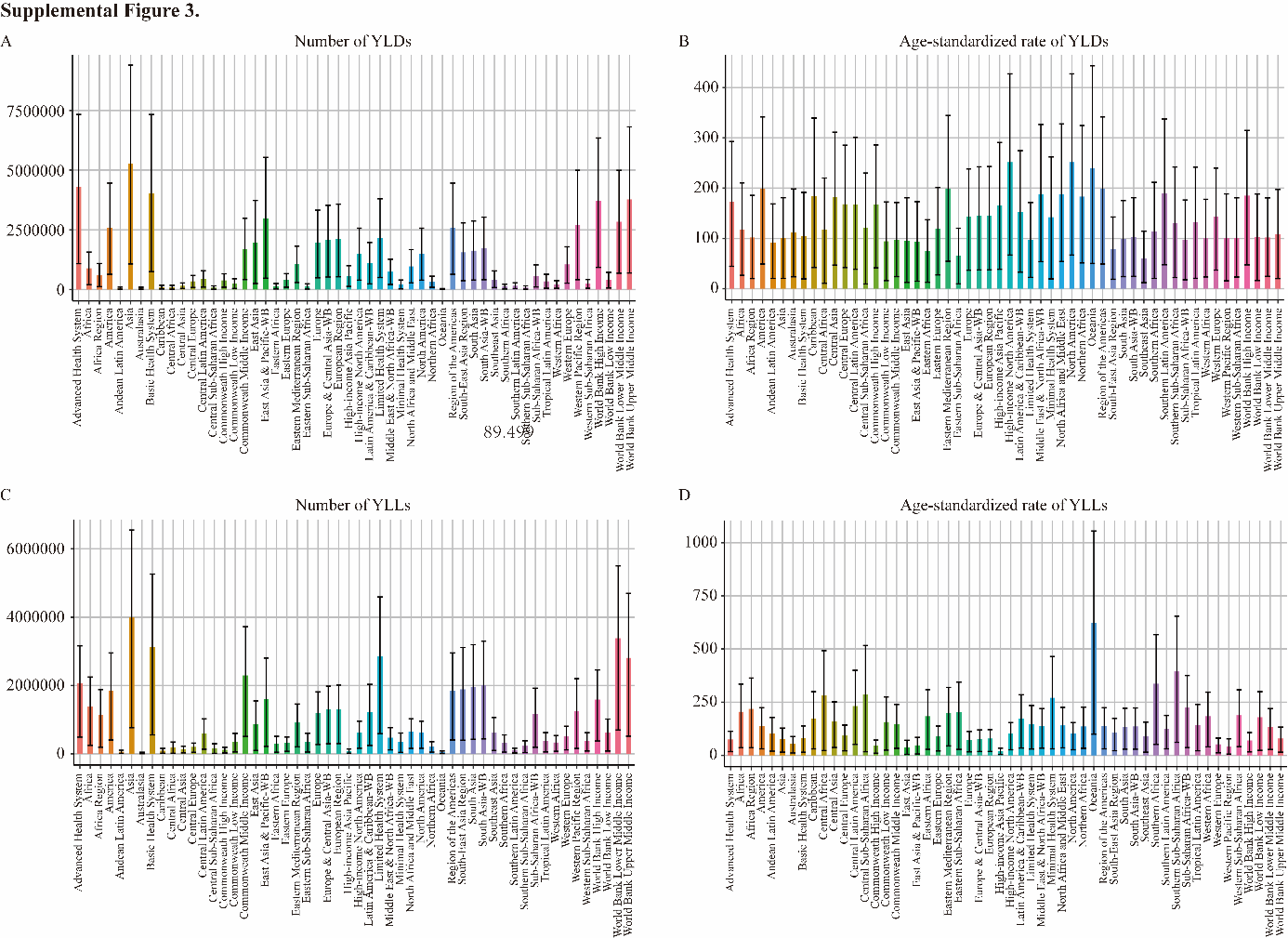


**Supplemental Figure 4. Predictions of Global Trend in YLDs and YLLs of T2DM due to Dietary Risk from 2030 to 2050.**


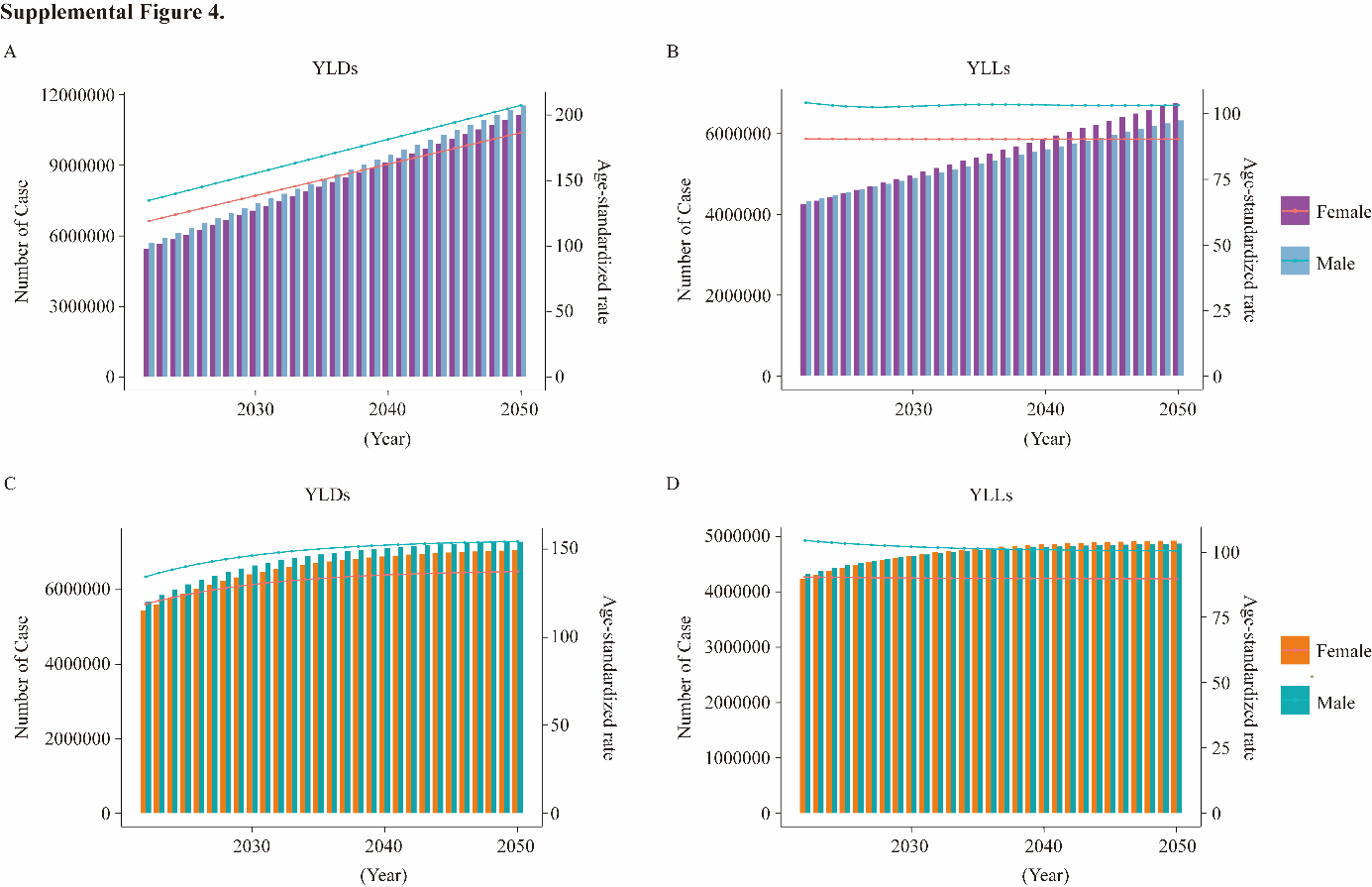

Supplement: Supplementary file 1 [file DataSheet1.docx]
